# *Schistosoma mansoni *infection reduces the incidence of murine cerebral malaria

**DOI:** 10.1186/1475-2875-9-5

**Published:** 2010-01-05

**Authors:** Judith H Waknine-Grinberg, Daniel Gold, Ariel Ohayon, Eliezer Flescher, Alina Heyfets, Michael J Doenhoff, Gabriele Schramm, Helmut Haas, Jacob Golenser

**Affiliations:** 1 Department of Microbiology and Molecular Genetics, The Hebrew University of Jerusalem, Jerusalem, Israel, 91120; 2 Laboratory of Membrane and Liposome Research, Department of Biochemistry, The Hebrew University of Jerusalem, Israel; 3 Department of Clinical Microbiology and Immunology, Sackler School of Medicine, Tel Aviv University, Israel; 4 Shraga Segal Department of Microbiology and Immunology, Ben-Gurion University of the Negev, Beer-Sheva, Israel; 5 The School of Biology, The University of Nottingham, Nottingham, UK; 6 Research Center Borstel, Borstel, Germany

## Abstract

**Background:**

*Plasmodium *and *Schistosoma *are two of the most common parasites in sub-tropical areas. Deregulation of the immune response to *Plasmodium falciparum*, characterized by a Th1 response, leads to cerebral malaria (CM), while a Th2 response accompanies chronic schistosomiasis.

**Methods:**

The development of CM was examined in mice with concomitant *Schistosoma mansoni *and *Plasmodium berghei *ANKA infections. The effect of S. *mansoni *egg antigen injection on disease development and survival was also determined. Cytokine serum levels were estimated using ELISA. Statistical analysis was performed using t-test.

**Results:**

The results demonstrate that concomitant *S. mansoni *and *P. berghei *ANKA infection leads to a reduction in CM. This effect is dependent on infection schedule and infecting cercariae number, and is correlated with a Th2 response. Schistosomal egg antigen injection delays the death of Plasmodium-infected mice, indicating immune involvement.

**Conclusions:**

This research supports previous claims of a protective effect of helminth infection on CM development. The presence of multiple parasitic infections in patients from endemic areas should therefore be carefully noted in clinical trials, and in the development of standard treatment protocols for malaria. Defined helminth antigens may be considered for alleviation of immunopathological symptoms.

## Background

Malaria, an infectious disease caused by the *Plasmodium *parasite, is a source of enormous morbidity and mortality. Cerebral malaria (CM), seen in about 7% of *Plasmodium falciparum *malaria cases, is characterized by the presence of neurological features, especially impaired consciousness [1]. The simplified explanation for CM pathogenesis is adherence and sequestration of parasitized erythrocytes, immune cells and platelets to vascular endothelial cells lining the small blood vessels of the brain. Thus, parasite-triggered cerebral inflammation is a possible cause of death from CM [2]. The immune response is critical in determining the outcome of infection [2]. CM is characterized by a Th1 response, with overproduction of some cytokines (e.g. interferon-γ, IFNγ), combined with underproduction of others (e.g. interleukin-10, IL-10) [3].

By analogy with the inhibition of autoimmune disease development by helminthic infections [4], it has been demonstrated, both in murine studies and in humans, that concomitant helminth infection may change the course of *Plasmodium *infection [5,6].

Schistosomes are parasitic trematodes found in subtropical and tropical areas. Among human parasitic diseases, schistosomiasis ranks second behind malaria in terms of socio-economic and public health importance. In many areas of the world, schistosomiasis and malaria are co-endemic: shared antigens and cross-reactive antibodies to different components of the two parasites have been detected [7]. Chronic helminthic infections are established through modulation of the host immune system. In schistosomal infection, each pair of male and female worms produces hundreds of eggs per day; egg-associated glycolipids and glycoproteins are the main target of the host humoral immune response [8]. In schistosomiasis, the early phase of infection is characterized by Th1 immune responses, which progress to a Th2 response. This pattern of cytokine expression is also found in non-cerebral (severe anaemic) malaria. In contrast, CM results from a predominantly Th1 response [9]. In schistosomiasis, the Th2-type responses are driven by schistosome egg antigens (SEAs) with intact carbohydrate moieties [10]. A major secretory glycoprotein, the IL-4-inducing principle from schistosome eggs IPSE (also known as alpha-1, or IPSE/alpha-1) [11], was identified as the bioactive component in *Schistosoma mansoni *egg extracts [12]. IPSE/alpha-1 triggers basophils to release IL-4, leading to subsequent IL-13 and additional IL-4 expression, the latter cytokine being a potential key player in Th2 biasing [8].

Several studies in murine models demonstrate a non-consistent effect of *Schistosoma *infection on malaria development, which is mostly expressed as enhancement of parasitaemia [13]. Similar conclusions concerning human malaria are based on study cases in endemic areas [14]. This research examined the effect of a pre-existing schistosomal infection on the development of murine CM, and the possible role of a main schistosomal antigen, IPSE/alpha-1, in the effect seen. The results demonstrate that IPSE/alpha-1 has a role in changing the course of malaria infection, leading to increased survival, and that a shift in cytokine expression is associated with CM reduction. Overall, this research demonstrates that CM may be alleviated by schistosomiasis.

## Methods

### Parasites

*Schistosoma mansoni *cercariae and schistosomula: an Egyptian strain of *S. mansoni*, kept in Puerto Rican *Biomphalaria glabrata *snails and ICR mice, was used throughout this work. Cercaria shedding was induced by subjecting infected, water-immersed snails to light for 1.5 hours. The cercariae were concentrated by cooling and low speed centrifugation.

The ANKA strain of *Plasmodium berghei *(MRA-311, CDC, Atlanta) was maintained in vivo by serial transfer of parasitized erythrocytes from infected to naive mice.

### Hosts

ICR HSD (Harlan-Sprague-Dawley) male mice aged six to seven weeks were used in all experiments; eight to 10 mice per group. The mice were housed under standard light and temperature conditions and were provided with unlimited access to water and food. The experiments were carried out in accordance with institutional guidelines for animal care, by a protocol approved by the Animal Ethical Care Committee of The Hebrew University of Jerusalem, AAALAC (Association for Assessment and Accreditation of Laboratory Animal Care) accreditation number #1285. The choice of genetically heterogenous mice was made in order to enable a more accurate reflection of CM susceptibility and the possible effect of coinfection in human beings. The validity of the CM model in outbred mice has previously been demonstrated [15]

### Experimental setup

Mice were infected with 50 or 100 *S. mansoni *cercariae by subcutaneous injection. Four or seven weeks later, one group of each condition was infected with 5 × 10^4 ^*P. berghei*-parasitized red blood cells, an inoculum which leads to cerebral malaria in the majority of infected mice. Additional control groups consisted of mice infected with *P. berghei *or *S. mansoni *only. Worm burdens were determined by dissection of *Schistosoma*-infected mice. Alternatively, mice were first infected with *P. berghei *and subsequently administered complete *S. mansoni *egg extract (SmEA) [12], egg extract from which IPSE/alpha-1 was removed (SmEA ΔIPSE/alpha-1) [16] (45 μg/mouse/day on days -7, 0, and 3 post-infection; 22.5 μg/mouse on day 6 post-infection), or IPSE/alpha-1 [8,11] (5 μg/mouse/day from day -7 to +4 post-infection), by intravenous (iv) administration. Parasitaemia was monitored every other day by thin blood smears prepared from tail blood. These were stained with a Giemsa solution and examined under a light microscope. Blood samples were taken for subsequent cytokine analysis by ELISA (Biolegend, Israel). The minimum detectable concentrations were 4 pg/ml, 2 pg/ml, 1 pg/ml, and 30 pg/ml for IFNγ, TNF, IL-4, and IL-10, respectively.

**Figure 1:**
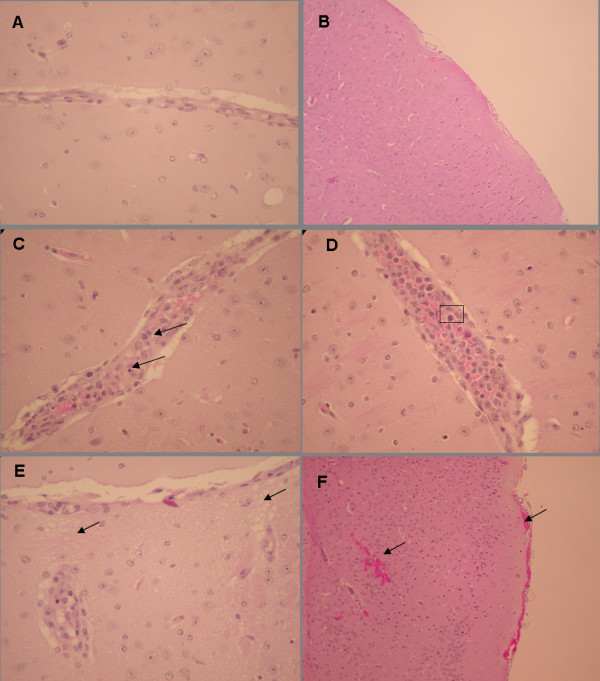
**Brain histology of non-infected and PbA-infected ICR mice**. Brains were removed from control non-infected mice (n = 10) or PbA-infected moribund mice (n = 10) on day 8 post- PbA inoculation. Representative sections are shown for each group. (A) Representative section of the cortex of a control mouse, demonstrating lack of vessel distention or pathology (magnification 20×). (B) Representative section of the submeningeal cortex of a control mouse, demonstrating the lack of haemorrhages or cellular infiltrations (magnification 10×). (C) Mononuclear cell aggregation in a meningeal vessel, increased cellularity, and piknotic nuclei in a prostrate mouse (magnification 20×). (D) Distended thallamic blood vessel; mononuclear cell interacting with activated endothelial cells (boxed) in a prostrate mouse (magnification 40×). (E) "Spongy" tissue - evidence of edema in a PbA-infected mouse suffering from coma (magnification 40×). (F) Meningeal haemorrahges in a PbA-infected mouse suffering from coma (magnification 10×).

The link between immune responses, death and cerebral malaria in mouse models has previously been demonstrated [2]. Observed clinical symptoms, including an accelerated drop in temperature and death at low parasitaemia, indicate CM [17]. Parasitaemia levels, the appearance of neurological symptoms and changes in weight, temperature, and haematocrit of infected mice were evaluated. Clinical signs, which appear one to two days before death from CM, include marked coat staring, hunching, wobbly gait and reduced locomotion, convulsions, and coma. Brain pathology observed in mice dying of CM includes haemorrhages, mononuculear cell infiltrations and the development of brain oedema (Figure 1). Mice that died at a parasitaemia of up to 15% with accompanying neurological symptoms, drastic weight reduction, and a body temperature of 34°C or below were considered to have died of CM. Mice which did not die from CM died from severe malaria-induced anaemia and high parasitaemia, as has been reported in all other cases where mice do not succumb to *P. berghei*-induced CM [18].

### Histology

Brains of non-infected and PbA-infected moribund mice were taken for histological examination. Mice were deeply anaesthetized and sacrificed by intracardial perfusion with 10 ml ice-cold PBS. Brains were removed, fixed in a 4% formaldehyde solution, and embedded in paraffin. Paraffin-embedded tissues were cut into 5 μm slices, deparaffinated, and stained with haematoxylin and eosin (H&E) before coverslipping, according to standard procedure.

### Statistics

p values were calculated using Students t-test.

## Results

**Figure 2:**
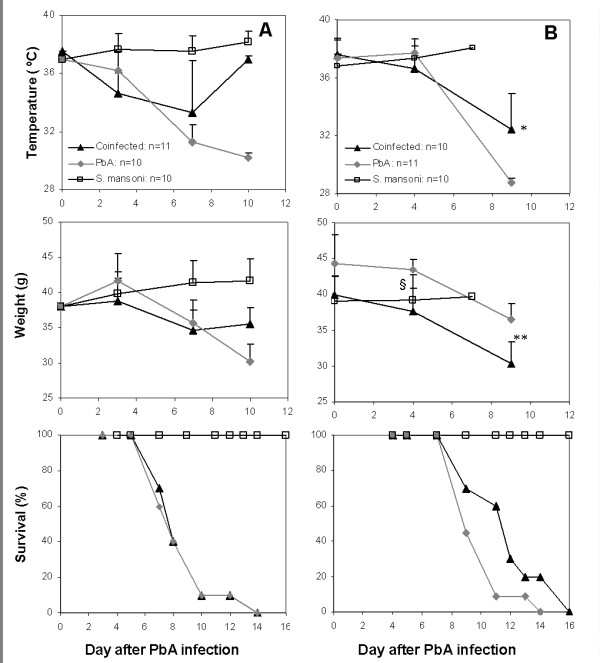
**Changes in temperature and body weight, and survival of mice infected with PbA 4 weeks (A), or 7 weeks (B) after *S. mansoni *infection, and in corresponding PbA- and *S. mansoni *infected control mice**. Weight and temperature were monitored until days 9-11, when most of the deaths in the PbA-infected groups occurred. Error bars represent SD. *p < 0.001 (t-test), co-infected vs. *S. mansoni *and PbA-infected groups; ^§^p < 0.0001 (t-test), PbA- vs. co-infected mice; **p < 0.002 (t-test), co-infected vs. PbA-infected mice.

Two different infection schedules were examined, in order to determine how long after worm infection a protective effect against CM may be seen. Mice were divided into four groups, two of which were infected with *P. berghei *four or seven weeks after injection of 100 *S. mansoni *cercariae. The remaining two groups served as controls for *S. mansoni *or *P. berghei *infection. In mice co-infected four weeks post-*S. mansoni *infection, no significant effect was seen on body temperature, weight loss (Figure 2A), or CM (60% in co-infected vs. 70% in *P. berghei*-infected mice). In contrast, when mice were co-infected seven weeks after cercariae injection, a protective effect of the pre-existing *S. mansoni *infection was seen: co-infected mice displayed lower rates of CM (30% vs. 60% in control mice), correlated to higher body temperatures and weights, and a delay to death (Figure 2B). The haematocrit of co- and *S. mansoni*- infected mice was similar (an average of 53 and 54 on day 3; 47 and 49 on day 7 after *P. berghei *infection, respectively), while the haematocrit of *P. berghei*-infected mice dropped from an average 51 on day 3 to 39 on day 7 after infection. Worm loads in the *S. mansoni *control group were examined four or seven weeks post-infection. Worm loads were not detectable four weeks after cercariae injection. Worm loads in mice co-infected seven weeks post-*S. mansoni *infection were found to be slightly lower than in mice infected with *S. mansoni *only (17.6 ± 2.3 vs. 22.6 ± 7.3, worms, respectively). Equal numbers of male and female worms were seen in each mouse.

**Figure 3:**
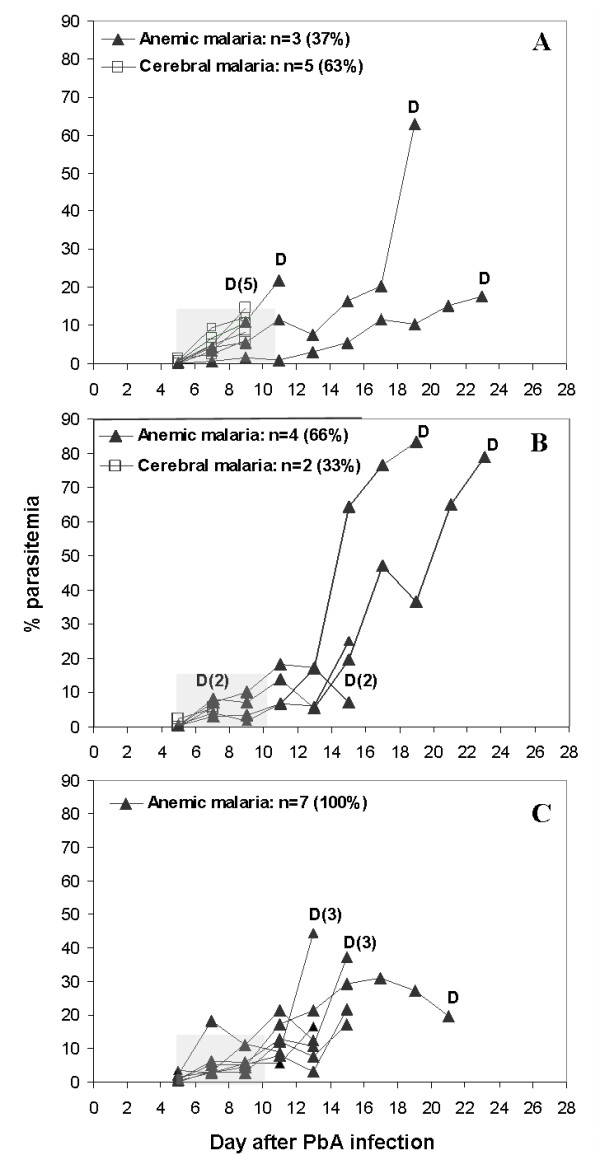
**The effect of *S. mansoni *infection on the development of cerebral malaria in ICR mice**. Mice infected with 5 × 10^4 ^PbA-infected erythrocytes (**A**), 5 × 10^4 ^PbA-infected erythrocytes, seven weeks after injection of 50 *S. mansoni *cercariae (**B**), or 5 × 10^4 ^PbA-infected erythrocytes, seven weeks after injection of 100 *S. mansoni *cercariae (**C**). Each line represents one mouse; D notes mouse death. Co-infection of mice with 100 cercariae caused a significant reduction in cerebral malaria (p < 0.0001, t-test). Shaded areas denote the period of CM susceptibility and death.

The next experiment was planned to evaluate the effect of cercarial dose on the malarial infection. As shown (Figure 3), *P. berghei *infection led to CM in five of eight control mice (63%); the remaining mice died of severe anaemic malaria. Two of six mice (33%) co-infected with 50 cercariae died of CM. Infection with 100 cercariae, however, prevented cerebral malaria in all 7 co-infected mice (Table 1, Experiment 1, 100 cercariae). No significant differences in parasitaemia were seen during the first 11 days of infection, the time period in which death from CM occurs. On day 7 post-infection, the average haematocrit in the co-infected group was 49 ± 9, compared to 39 ± 3 in the *P. berghei*-infected group and 49 ± 3 in the *S. mansoni *group, indicating the absence of severe anaemia. A delay in time of death was observed in the *S. mansoni*-infected groups relative to the control group: on day 13 post-infection the majority of control mice were dead, whereas 66% of mice injected with 50 cercariae, and all of the mice injected with 100 cercariae were still alive. On day 9, *P. berghei*-infected mice had lost an average 15 ± 2% of their initial body weight, while co-infected mice lost 24 ± 5%. This result may be attributed to the high parasite burden in the co-infected mice. In comparison, mice infected solely with *S. mansoni *gained an average 2 ± 6% body weight during this period. None of the mice infected solely with *S. mansoni *died, indicating that pathology due to the helminth infection was not a cause of death in these experiments. Each experiment was repeated (Table 1). Overall, the results indicate that *S. mansoni *infection provides protection against CM.

**Table 1:** The effect of *S. mansoni *infection on the development of cerebral malaria.

**50 cercariae**	***P. berghei*-infected mice**		Co-infected mice*	
	**Cerebral malaria**	**Anaemic malaria**	**Cerebral malaria**	**Anaemic malaria**
Experiment 1	5	3	2	4
Experiment 2	7	3	2	9
Total	12/18	6/18	4/17	13/17
(%)	(67%)	(33%)	(24%)	(76%)
				
**100 cercariae**	***P. berghei*-infected mice**		Co-infected mice*	
	**Cerebral malaria**	**Anaemic malaria**	**Cerebral malaria**	**Anaemic malaria**
Experiment 1	5	3	0	7
Experiment 2	7	3	3	7
Experiment 3	6	5	2	8
Total	18/29	11/29	5/27	22/27
(%)	(62%)	(38%)	(19%)	(81%)

* Mice were infected with 5 × 10^4 ^*P. berghei*-parasitized erythrocytes seven weeks after injection of 50 or 100 *S. mansoni *cercariae.

**Figure 4:**
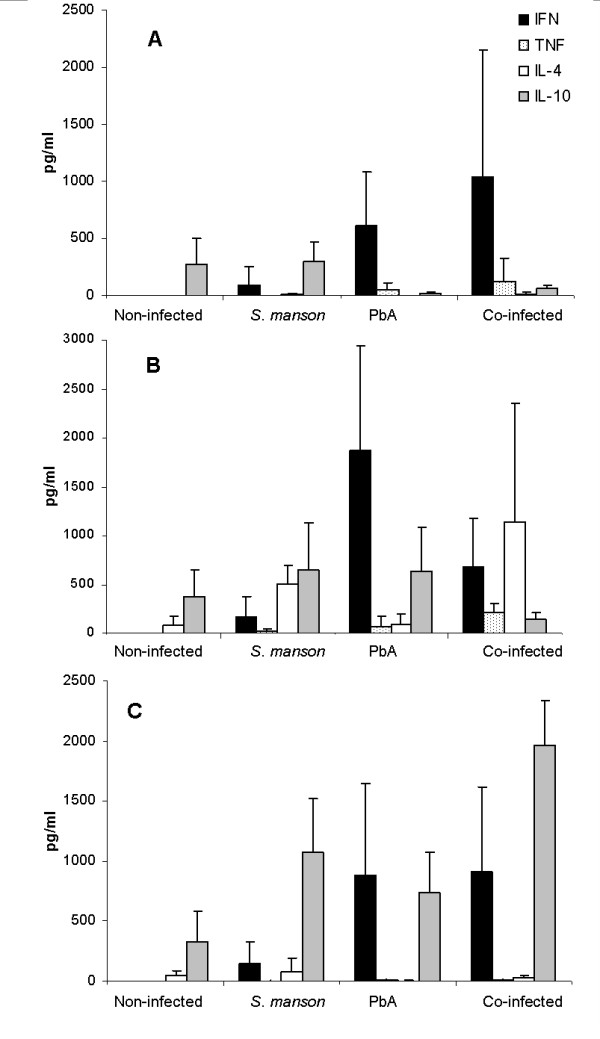
**Average serum cytokine levels (pg/ml) ± SD in infected and non-infected ICR mice (n = 5 at each time point)**. Mice were infected with PbA 7 weeks after *S. mansoni *infection. Cytokine levels were measured on day 1 (**A**), day 7 (**B**), and day 10 post-PbA infection (**C**).

Serum cytokine profiles were determined at several time points in mice co-infected with *P. berghei *seven weeks after injection of 100 *S. mansoni *cercariae (days 1, 7, and 10 post-*P. berghei *infection). Control groups included non-infected mice and mice infected with *S. mansoni *or *P. berghei *only (Figure 4). On day 1, an increase in serum IFNγ was seen in *P. berghei*infected mice (p < 0.05), while no significant increase in IFNγ levels was seen in co-infected mice. IFNγ levels were below detection in non-infected and *S. mansoni*-infected mice. Although TNF levels rose slightly in the PbA- and co-infected groups, this change was not statistically significant. Co-infected mice displayed higher IL-4 relative to both non-infected and PbA-infected mice (p < 0.05). IL-10 levels were similar in *S. mansoni*- and non-infected mice; co-infected mice displayed higher IL-10 compared to *P. berghei*-infected mice (p < 0.05). The difference in IL-10 levels between PbA- and *S. mansoni*-infected mice was significant (p < 0.01). By day 7, IFNγ levels had risen significantly in *P. berghei*- and co-infected mice, compared to non-infected mice (p < 0.05) and *S. mansoni*-infected mice (p < 0.01). Although no statistically significant differences in IFNγ were seen when comparing PbA- and co-infected mice, the difference in TNF was significant (p < 0.05). Co-infected mice showed a dramatic rise in IL-4 (p < 0.05); this rise in serum IL-4 (day 7 compared to day 1, corresponding to days 42 and 36 of *S. mansoni *infection) is presumably due to an egg-induced Th2 response. IFNγ was higher in *P. berghei*- and co-infected mice compared to the S. *mansoni*-infected group (p < 0.01, p < 0.05, respectively), and TNF was raised in co-infected mice compared to the S. *mansoni*-infected group (p < 0.05). These differences are presumably due to the ongoing inflammatory response caused by the malaria parasites. On day 10, no significant differences in IFNγ were seen when comparing PbA- to *S. mansoni*-or co-infected mice, or between *S. mansoni*- and co-infected mice. Differences in IL-10 were significant (p < 0.05) when comparing co-infected and *S. mansoni*- or PbA-infected mice. TNF levels were higher in co-infected vs *S. mansoni*-infected mice. No significant differences in IL-4 were seen between the groups.

**Figure 5:**
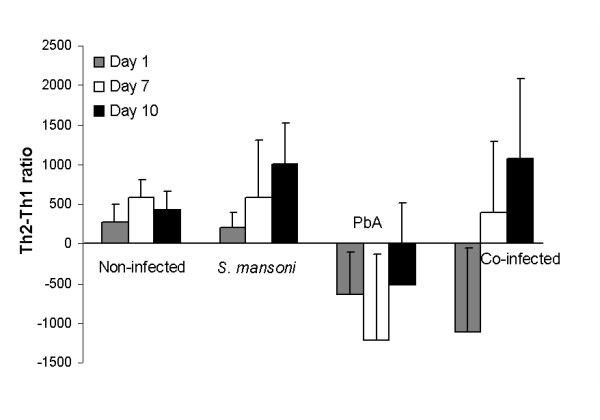
**Average Th2-Th1 ratio**. The balance between Th1 and Th2 responses was determined using the cytokine levels of each mouse, as follows: Th2/Th1 ratio = (sum of IL-4 and IL-10 levels) - (sum of IFNγ and TNF levels). Average values ± SD are presented per day. p < 0.05 (t-test) when comparing *non-infected and *S. mansoni*-infected mice; **non-infected and PbA-infected mice; ^†^*S. mansoni*- and PbA-infected mice; ^‡^*S. mansoni*- and co-infected mice; ^§^PbA- and co-infected mice.

The overall immune response in the different groups may be summarized using the ratio between the Th1- and Th2-group cytokines examined (Figure 5). IL-4 and IL-10 are signature Th2 cytokines; IL-4 is critically involved in biasing the immune reaction toward a Th2 phenotype. In mice which develop severe anaemic malaria, a transient early rise in IFNγ typically occurs, while in mice which develop CM, IFNγ levels remain high as the result of immune deregulation [2]. The results show that *P. berghei *infection caused an immediate, significant shift in the Th response of infected mice: both *P. berghei*-infected and co-infected groups showed skewing towards a Th1-type response when cytokine ratios were compared to non- or *S. mansoni*-infected mice (p < 0.01, p < 0.05, respectively). However, on day 7, *P. berghei*-infected mice still showed a clear Th1 shift, compared to non-infected and *S. mansoni*-infected mice (p < 0.01); co-infected mice displayed a prevalently Th2 response (p < 0.05). On day 10, the difference in Th1 response in *P. berghei*- vs. *S. mansoni*-infected mice was still apparent (p < 0.05), but no difference was seen when comparing *P. berghei*- and co-infected mice. Overall, the results hint at a Th2 shift caused by the pre-existing schistosomal infection.

**Figure 6:**
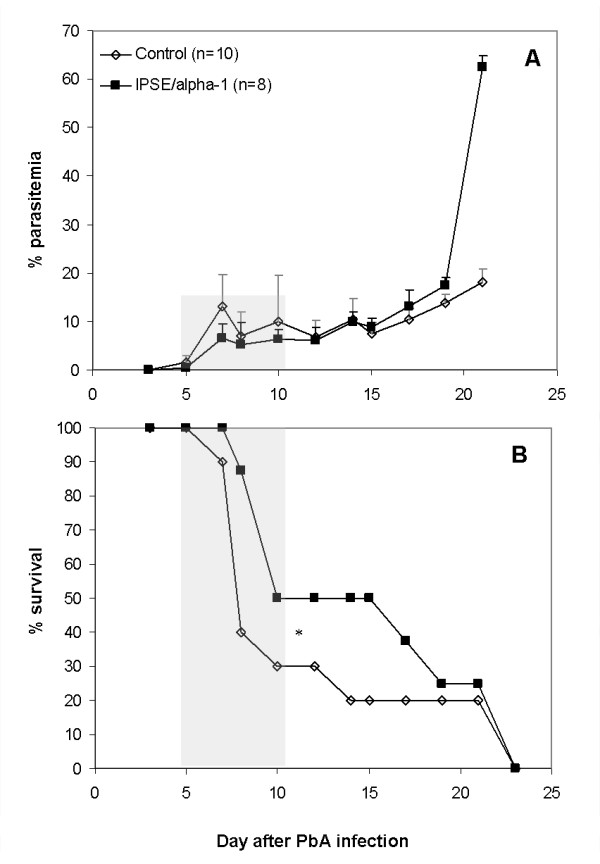
**Parasitaemia (A) and survival (B) of mice administered IPSE/alpha-1 by i.v. injection from day 4 before PbA infection to day 7 post-infection**. Control mice were injected with PBS. *Significant difference in delay to death (p = 0.005, t-test). Shaded areas denote the period of CM susceptibility and death.

**Figure 7:**
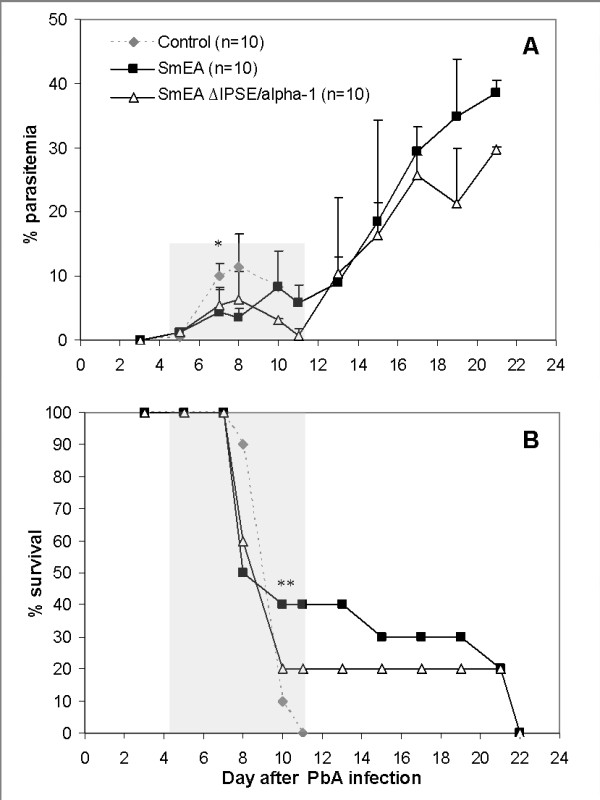
**Parasitaemia (A) and survival (B) of control mice (n = 10) and mice administered complete *S. mansoni *egg extract (SmEA; n = 10) or egg extract from which IPSE/alpha-1 was removed (SmEA ΔIPSE/alpha-1; n = 10), by i.v. injection, on days 7 and 0 before PbA infection and days 3 and 6 post-infection**. Control mice were injected with PBS. *Parasitaemia was slightly lower in mice treated with SmEA or SmEA ΔIPSE/alpha-1 compared to control mice, on days 7-10 post-infection (p < 0.05, t-test). **A significant delay to death was seen between control and treated mice (p < 0.05, t-test), but not between the treated groups (p = 0.2, t-test). Shaded areas denote the period of CM susceptibility and death.

Our next step was to examine the effect of IPSE/alpha-1 administration and injection of intact or IPSE/alpha-1-depleted egg extract, on the course of *P. berghei *infection. Two protocols were used to determine the effect of IPSE/alpha-1 on CM. In the first experiment, 5 μg/mouse IPSE/alpha-1 was injected i.v. from day -4 to day +7 post-infection. Although antigen injection did not improve the rate of CM (50% of control and 75% of treated mice; Figure 6A), a significant delay in death was seen. The median survival was 15 days among treated mice, compared to 8 days in the control group (Figure 6B). In order to verify the role of IPSE/alpha-1 on the course of the disease, mice were injected with SmEA or SmEA ΔIPSE/alpha-1. Both extracts were administered by i.v. injection at a dose of 45 μg/mouse on days -7, 0 and +3 followed by 22.5 μg/mouse on day +6 post-infection. Administration of SmEA had a slight effect on CM development, as did SmEA ΔIPSE/alpha-1 injection (Figure 7), with the rate of CM dropping from 90% in the control group to 70% and 80%, respectively. As in the previous experiments, IPSE/alpha-1 delayed mouse death. The complete extract was more effective than the extract lacking IPSE/alpha-1 (Figure 7).

## Discussion

In malaria-endemic areas, infections caused by intestinal, schistosomal or filarial parasites commonly coexist with malaria in the same patient [19,20]. There has been much speculation as to an immunological aspect of the effect of schistosomes, which induce a host Th2 response [21], on malaria parasites.

In the present study, we investigated the effect of schistosomal parasites or their antigens on the development of the Th1-associated cerebral malaria, using the established procedure for murine CM induction. As *S. mansoni *affects mainly the liver, and *Plasmodium *infects hepatocytes prior to infection of the red blood cells, a more accurate experimental setup would have been to use the full plasmodial life cycle, i.e. starting from infection of the mice by the bite of infected mosquitoes. In case of a full-cycle infection, the Th2 response to schistosome eggs would likely extend to sporozoite- and liver- stages of plasmodia. In analogy, it has been demonstrated that pre-treatment to tip the cytokine balance towards a Th1 response significantly reduces the malaria parasite load in the liver [22]. Thus, we suppose that the schistosomes would most likely affect the development of plasmodial sporozoites via local interactions in the liver as well as general immunomodulation.

No significant difference in CM rate or in survival was seen when comparing *P. berghei*-infected and co-infected mice 4 weeks post-*S. mansoni *infection. In contrast, concomitant infection seven weeks post-cercariae injection caused a marked reduction in CM. The lack of effect in the first case may be explained by the fact that no mature worms are seen by four weeks after cercariae injection. During the prepatent period of *S. mansoni *infection, the first 4-5 weeks following exposure to cercariae, the immune response is primarily Th1 in nature, while an egg antigen-specific Th2 response is seen by eight weeks post-infection [23]. IFNγ production is essential for CM development [2,17]. Accordingly, each stage of schistosomal infection may affect the development of CM in a different manner: we assume that the initial Th1 response caused by the worms prevented any ameliorating effect. During the patent phase of schistosomal infection (7 weeks after injection of cercariae), a decreased rate of cerebral malaria was accompanied by higher body temperature and less severe weight reduction. Analysis of cytokine levels revealed that the pre-existing *S. mansoni *infection caused a Th2 shift, which translated to the improved disease profile. This shift is probably the result of an overall change in the balance of cytokines, chemokines and adhesion molecules which participate in the pathogenesis of CM. Therefore, an estimate of a several factors (e.g. a sum of Th1 vs. Th2 cytokines) will better predict and correlate to the clinical situation.

Additional cytokine analysis 4 weeks after *S. mansoni *infection may demonstrate that the Th1 to Th2 shift has not yet occurred. However, at this stage eggs are not yet deposited. As mature egg, deposition is the major stimulus for the production of Th2 cytokines in murine *S. mansoni *infection [24], this is the reason for our experimental design. The effect of co-infection on the rate of CM was reduced when mice were injected with a lower number of cercariae (50 vs.100), presumably because of the less pronounced immune response expected to have been elicited upon helminth infection.

Egg antigens are crucial in the pathology caused by schistosomes [10,25]. In an established *S. mansoni *infection, egg-laying by the adult female worms causes a Th2 shift in the host immune response. SmEA is a complex mixture of both structural and secretory egg antigens, the latter of which are thought to react primarily with host immune cells [26]. IPSE/alpha-1, a secretory glycoprotein present only in mature eggs, is a general activator of both human and murine basophils, triggering their degranulation and IL-4 production as well as the release of IL-13 and histamine [12,15]. Depletion of IPSE/alpha-1 from SmEA completely abrogates the SmEA-induced basophil activation [12]. The immunogenicity of IPSE/alpha-1 is due in part to the presence of glycans that carry one or more Lewis X motifs, which, in the context of glycan conjugates, induce production of Th2-associated mediators such as IL-10 and prostaglandin E2 [8]. Egg glycolipid and glycoprotein epitopes found in both adult and larval schistosomes are the predominant targets of the humoral immune response in schistosomiasis [8]. The activity of IPSE/alpha-1 is independent of basophil preconditioning due to prior infections, and IL-4 production can be induced in basophils in the liver, a major site of *S. mansoni *egg deposition [27].

In our experiments, injection of IPSE/alpha-1 caused a consistent delay in mouse death compared to non-treated mice, and a slight reduction in parasitaemia during the first week of infection (p < 0.05). In contrast to *S. mansoni *infection, no significant reduction in CM was induced by injection of SmEA or IPSE/alpha1. Delay in death, however, was pronounced upon injection of SmEA, an effect which was reduced upon injection of SmEA ΔIPSE/alpha-1 suggesting a role of IPSE/alpha-1. Whether the prolonged survival of the *P. berghei*-*S. mansoni *co-infected mice is due an anti-inflammatory effect of IPSE/alpha-1-induced IL-4 (and IL-13) remains to be determined. Nevertheless, this is an obvious assumption, because apart from the direct polarization of naïve T helper cells towards the Th2 phenotype, IL-4 has been shown to have a variety of anti-inflammatory effects, such as inhibition of the LPS-induced release of IL- 6, TNF, and IL1-β from monocytes [28], inhibition of Th17-development [29] and together with IL-13 the alternative activation of macrophages [30]. The observation that schistosome infection was superior to administration of SmEA or IPSE/alpha-1 in preventing CM may be explained by the continuous flow of egg antigens during infection *versus *bolus application in the experimental set-up and/or additional immunomodulatory factors released during active infection.

It should be emphasized that the result of infection in a malaria-endemic area has a complex pattern due to the variety of pathogens and the genetic variability of the population. Despite this complexity, various investigations have provided clues regarding the effect of concomitant helminthic infection on the course of malaria. Observations in Thailand showed that co-infection with intestinal helminths increased *P. falciparum *incidence two-fold but decreased CM by 64% [31]. *Schistosoma haematobium *infection was shown to reduce malaria severity by increasing Th2 responses in *P. falciparum*-infected children [32]; a recent ex-vivo study indicates that helminth infections cause an increase in IL-10 but no change in TNF [33], indicating suppression of pro-inflammatory responses. Age infection profiles indicate that school-age children are at the highest risk of co-infection [34]; underlying schistosomiasis was associated with protection against clinical falciparum malaria in children under the age of 9 [35]. The effects of schistosomal infection were shown to be dependant on egg load as well as age. Plasmodial levels in children with light egg loads were lower when compared to children with no schistosomal infection [35], while synergy was observed in the case of high egg loads [36]. In mice, existing *S. mansoni *infection interfered with attempts to vaccinate against BCG [37] or HIV components [38]. Intestinal helminths have also been associated with protection from immunopathology. Nacher et al [39] indicate protection from CM in human infection with intestinal helminths. In a murine model, CBA/J mice inoculated with *Brugia pahangi *developed a Th2 immune response, and, as a result, a lower rate of CM following *P. berghei *injection [40]. In contrast, Bejon *et al *[20] could not demonstrate an effect of intestinal helminths on *P. falciparum *infection. It should be noted that although these studies addressed patient exposure to both *Plasmodium *and helminths, additional infections, a factor shown by murine models to be possibly significant [41], was not investigated.

The effect of co-infections (schistosomes, hookworms, and plasmodia) on anaemia should also be considered in view of competition between haematopoietic and immunological compensations [42]. In addition, the concentration of free haem, which is also affected by the activity of these parasites, is related to the expression of haem oxygenase, which prevents CM [43]. Notably there can be significant changes in the vasculature induced by schistosome infection including the development of collateral circulation, the development of portal hypertension and hepato- and splenomegaly. All may have important consequences for blood flow and for malaria parasite distribution in concomitantly infected hosts. The impact of schistosome infection on anaemia may also be important. Red cell production dynamics and the balance between mature erythrocytes and reticulocytes may have been altered by the presence of adult erythrocyteconsuming schistosomes.

## Conclusions

The results of this research demonstrate that a preexisting schistosome infection may drastically affect the outcome of *Plasmodium *infection. Protection from CM appears to be a function of *S. mansoni *parasite load. As mentioned, microbial and parasitic infections in malaria-endemic areas are not controlled and it is likely that many persons are infected by more than one parasite species (including helminths). Presence or absence of a pre-existing infection may explain why certain individuals develop cerebral malaria while others exhibit minor symptoms only. The presence of multiple parasitic infections in patients from endemic areas should, therefore, be carefully noted in future clinical trials, and in the development of standard treatment protocols for malaria infection.

## Competing interests

MJD is the owner of BioGlab Ltd, which sells schistosome-derived material. SmEA was supplied by BioGlab for this work on a not-for-profit basis.

The authors declare that they have no competing interests.

## Authors' contributions

JHWG participated in the design and performance of the experiments, and in the writing of the manuscript. HH, MJD and GS participated in the design of the study, production of the schistosomal antigens and writing the manuscript. DG participated in the design, performance of the in vivo experiments using *S. mansoni*, and in critical reading of the manuscript. AO participated in cytokine measurements. EF and AH participated in experiments examining plasmodial development in mice. JG initiated and coordinated the study.

All authors have read and approved the final manuscript.
